# Effects of *Gasterophilus pecorum* infestation on the intestinal microbiota of the rewilded Przewalski’s horses in China

**DOI:** 10.1371/journal.pone.0251512

**Published:** 2021-05-11

**Authors:** Dini Hu, Yuzhu Chao, Boru Zhang, Chen Wang, Yingjie Qi, Make Ente, Dong Zhang, Kai Li, Kai Meng Mok

**Affiliations:** 1 School of Ecology and Nature Conservation, Beijing Forestry University, Beijing, China; 2 Qinhuangdao Forestry Bureau, Qinhuangdao, China; 3 Altay Management Station of Mt. Kalamaili Ungulate Nature Reserve, Altay, China; 4 Xinjiang Research Centre for Breeding Przewalski’s Horse, Urumqi, China; 5 Department of Civil and Environmental Engineering, University of Macau, Macao, China; University of Maine, UNITED STATES

## Abstract

Horse botflies have been a threat to the Przewalski’s horses in the Kalamaili Nature Reserve in Xinjiang of China since their reintroduction to the original range. As larvae of these parasites could infest the intestine of a horse for months, they could interact with and alter the structure and composition of its intestinal microbiota, affecting adversely its health. Nonetheless, there are no such studies on the rewilded Przewalski’s horses yet. For the first time, this study characterizes the composition of the intestinal microbiota of 7 rewilded Przewalski’s horses infected severely by *Gasterophilus pecorum* following and prior to their anthelmintic treatment. Bioinformatics analyses of the sequence data obtained by amplicon high throughput sequencing of bacterial 16S rRNA genes showed that *G*. *pecorum* infestation significantly increased the richness of the intestinal microbial community but not its diversity. *Firmicutes* and *Bacteroidetes* were found the dominant phyla as in other animals, and the parasitic infestation decreased the F/B ratio largely by over 50%. Large reduction in relative abundances of the two genera *Streptococcus* and *Lactobacillus* observed with *G*. *pecorum* infestation suggested possible changes in colic and digestion related conditions of the infected horses. Variations on the relative abundance of the genus groups known to be pathogenic or symbiotic showed that adverse impact of the *G*. *pecorum* infestation could be associated with reduction of the symbiotic genera *Lactobacillus* and *Bifidobacterium* that are probiotics and able to promote immunity against parasitic infection.

## 1. Introduction

The Przewalski’s horse (*Equus ferus przewalskii*) is a large and endangered wild equine ("EN"class) originally distributed in the Gobi Desert of Xinjiang, China and Mongolia [1,2]. It was extinct in its original range due to anthropogenic activities in the 1960s [3,4]. With only a few remaining in small captive breeding herds in Europe and North America [5,6], the Chinese government started in 1985 a reintroduction operation of the Przewalski’s horse according to the plan of the International Union for Conservation of Nature (IUCN), aiming to ultimately restore its wild population in China. Then Przewalski’s horses were transferred from the United States, Great Britain and Germany to breeding centers in Xinjiang and Gansu over a period of time [2,7]. Rewilding of them began in 2001 when the first batch of 27 bred Przewalski’s horses were released into the Kalamaili Nature Reserve (KNR) in Xinjiang [4,8]. Continuous monitoring of the released horses shows that they have been suffering from severe infestation of endoparasites of the *Gastrophilus* spp. or horse botfly [9–16]. It is known that larvae of horse botflies could infest the digestive tract of an equine for 8 to 10 months [17], causing lesions along the duodenal ampulla and the duodenum proximal segment [18], and can further induce some clinical diseases, such as anaemia, diarrhoea, gastric rupture, peritonitis, perforating ulcers, etc. [13]. Thus, the released Przewalski’s horses are captured annually for a short period of time in winter for parasitic infection assessment and corresponding expelling treatment. This exercise may in fact interrupt the rewilding process of the horses, but understanding the health status of the released horses is also important.

As larvae of horse botflies spend substantially amount of time in the stomach of an infected equine, their infestation could alter the landscape of the complex polymicrobial community of bacteria, viruses, archaea, fungi, and parasites in the gut of the host, and among all, the bacterial community plays the most important role in maintaining balance of the intestine environment [19,20]. It was shown that there are up to 10^15^ bacterial cells in the intestine of an equine with majority of them associated with degradation of non-digestible cellulosic and hemi-cellulosic forage [21]. A stable intestinal microbiota interacting with the host forms a mature immune system and supplies the needed nutrients to the host for its healthy survival [22]. Studies have shown that the distinguishable intestinal environment in mammal is closely related to the composition of its microbiota [23], hence identification of the core bacteria that define the enterotype of the intestinal microbiome is of importance [24,25]. For example, the intestinal microbes of vertebrates are mainly composed of *Firmicutes*, *Bacteroidetes*, *Actinomycetes*, *Proteobacteria* and *Clostridium* [26]. They are *Firmicutes* and *Bacteroidetes* for mammals [27], and *Firmicutes*, *Bacteroidete*s and some unclassified bacteria for herbivores [28]. However, stability of the intestinal microbiota can be disturbed by physical, chemical and biological factors internal and external of the host [29,30]. Some diseases such as grass sickness, colitis, and laminitis were found related to the dysbiosis of the intestinal microbiota in equids [31–33]. Meanwhile, cardiovascular disorders [34], inflammatory bowel disease [35], diabetes [36], rheumatoid arthritis [37], depression [38], and progression of cancer [39] were found associated with changes of intestinal microbial community in human. In mares, significant increase in relative abundance of *Proteobacteria* was found in their fecal microbiota prior to episodes of colic, while differences in membership and structure of their microbiota in those that developed large colon volvulus were observed [40].

Studies of the effects of parasitic infection on the intestinal microbiota of horses are still limited. A recent study on the interaction of strongyles and intestinal microbiota in two groups of Welsh ponies susceptible and resistant to parasitic infection [41] showed that the overall intestinal microbiota diversity and structure remained similar between the two groups during infection. Reduction of bacteria that may have contributed to disruption of mucosal homeostasis in the susceptible group was observed, while an increase in pathobionts together with changes in some predicted immunological pathways that are critical for the regulation of immune system and energy homeostasis were also observed in the susceptible group relative to the resistant one. Another study in the same period investigated the relationships between fecal egg counts of cyathostomins and intestinal microbial composition in a group of broodmares prior to and following anthelmintic treatment [42]. They found that the populations of *Methanomicrobia* and *Dehalobacterium* had an increasing trend in the group of broodmares with low parasitic fecal egg counts, meanwhile there was a significant reduction of the bacterial phylum TM7, and a transient expansion of *Adlercreutzia* spp. after anthelmintic treatment in the group of broodmares with high fecal egg counts. Both studies showed that relationships between parasitic infection and intestinal microbiome in horses are present, hence profiling of the intestinal microbiota in them could lead to potential biomarkers for parasitic infection. Profiling of the intestinal microbiota in Przewalski’s horses in China have been done before but mainly on the effects of their grazing sites (therefore their diet) and captivity. Li et al. (2019) [43] found that diet is an important factor that influences the structure of the intestinal microbiota of the horses while Tang et al. (2020) [44] suggested that captivity could decrease the diversity of their intestinal microbial community. However, there are no studies looking into the effects of parasitic infection on the intestinal microbiota of the Przewalski’s horses although infestation of the *Gastrophilus* spp. in them was found severe.

The aims of this study were (i) to assess the intensity of horse botfly infestation in the rewilded Przewalski’s horses, and (ii) to characterize the differences in relative abundance of bacterial taxa in their intestines with and without horse botfly infestation through anthelmintic treatment. Results of this study could provide good foundation for better understanding the impact of natural horse botfly infestation on the released Przewalski’s horses in the wild, which could help their long-term rewilding process and healthy survival.

## 2. Materials and methods

This study was carried out in accordance with the Chinese law, regulations of the Beijing Forestry University, and guidelines of animal research [45]. The experimental protocol was reviewed and approved by the Institution of Animal Care and the Ethics Committee of the Beijing Forestry University. The management authority of the Kalamaili Nature Reserve (KNR) in Xinjiang of China approved the collection of fecal and botfly larval samples from the Przewalski’s horses.

### 2.1 Fecal sample collection and parasitic larva enumeration

This study was carried out at the KNR from December 15th to 22nd, 2019. KNR (44°36’ ~ 46°00’ N, 88°30’ ~ 90°03’ E) is a desert steppe with its altitudes ranging from 600 m to 1470 m. It has an average annual temperature of 2.4°C, and its annual precipitation and evaporation are 160 mm and 2,000 mm, respectively. A group of Przewalski’s horses were driven from the wild in early November of 2019 into a fenced pasture of 1 km by 1.5 km, where they were fed with alfalfa. Sample collection started on December 15th by capturing, in random from the group, 7 adult Przewalski’s horses (4 male, 3 female) with similar body weight. They were kept individually in temporary enclosure of 8 m by 16 m in size. During their short captivity, the Przewalski’s horses were fed with alfalfa with plenty of water. Fresh fecal samples were first collected from them on the day of capture (Dec. 15th, 2019) before anthelmintic treatment. Each sample was sealed immediately into a sterilized centrifuge tube to avoid cross-contamination. These samples were primary for characterizing the intestinal microbial community of the Przewalski’s horses with parasitic infestation. After this initial fecal sample collection, each horse received an oral dose of ivermectin (Beijing Wanfeng Pharmaceutical Co. LTD, GMP (2006) 278) at 0.2 mg/kg of body weight for anthelmintic treatment, which could clear the infesting parasites within 2 to 3 days [11]. The mechanism of ivermectin is to inhibit directly the neurotransmission of invertebrate [46]. It is not recognized as an effective antibacterial compound [47]. There was no broad, large-scale impact on the gastrointestinal microbiota found in parasite-free horses after receiving anthelmintic treatment [48]. Hence changes in the intestinal microbiota of horses following anthelmintic treatment are mainly due to the removal of parasites, they could be used for parasitic infestation impact assessment. Feces discharged daily by each horse was collected for larva counting in the following 3 days (Dec. 16th– 18th, 2019) after its ivermectin administration. The larval samples were classified morphologically and counted by naked-eye inspection [16]. These larval samples were then stored in anhydrous ethanol. With the elimination half-life of ivermectin being about 3 days [49], fresh fecal samples were collected again from the horses on December 22nd, 2019 (7 days after ivermectin administration). These samples were for characterizing the intestinal microbial community of the Przewalski’s horses free of parasitic infestation. It is noted that all fecal samples were stored in liquid nitrogen immediately after collection. They were then transported back to the laboratory, and stored at -80°C until DNA extraction and 16S rRNA sequencing.

### 2.2 16S rRNA sequencing

#### 2.2.1 DNA extraction, PCR amplification and sequencing

Microbial DNA was extracted from fecal samples using the E.Z.N.A.® Soil DNA Kit (Omega Bio-tek, Norcross, GA, U.S.). The V4-V5 region of the bacterial 16S rRNA gene was amplified by PCR (95°C for 2 min, followed by 25 cycles at 95°C for 30 s, 55°C for 30 s, and 72°C for 30 s and a final extension at 72°C for 5 min) using primers 338F/806R, where the barcode was an eight-base sequence unique to each sample. The length of amplification was 468 bp. The PCR was performed in triplicate in a 20 μL mixture containing 4 μL of 5 × FastPfu Buffer, 2 μL of 2.5 mM dNTPs, 0.8 μL of each primer (5 μM), 0.4 μL of FastPfu Polymerase, and 10 ng of template DNA.

Amplicons were extracted from 2% agarose gels, purified using the AxyPrep DNA Gel Extraction Kit (Axygen Biosciences, Union City, CA, U.S.), and they were quantified using QuantiFluor™ -ST (Promega, U.S.). Purified amplicons were pooled in equimolar amounts and paired-end sequenced (2 × 250) on an Illumina MiSeq platform. The raw reads were deposited into the NCBI Sequence Read Archive (SRA) database (SRA accession number: SRS6587909, SRS6587915, SRS6587926, SRS6587934, SRS6587935, SRS6587910, SRS6587911, SRS6875831, SRS6875834, SRS6875835, SRS6875836, SRS6875837, SRS6875832, SRS6875833).

#### 2.2.2 Bioinformatics and statistical analyses

Raw fastq files were demultiplexed and quality-filtered using QIIME (version 1.9.1) with the following criteria: (i) 300 bp reads were truncated at any site receiving an average quality score <20 over a 50 bp sliding window, truncated reads shorter than 50 bp were discarded; (ii) reads not matching exact barcode, reads with a 2-nucleotide mismatch in primer sequences, and reads containing ambiguous characters were removed; (iii) only sequences with overlap longer than 10 bp were assembled according to their overlap sequence. Reads that could not be assembled were discarded. Operational taxonomic units (OTUs) were clustered with a 97% similarity cutoff using UPARSE (version 7.0.1090), and chimeric sequences were identified and removed using UCHIME. The taxonomy of each 16S rRNA gene sequence was analyzed by RDP Classifier (http://rdp.cme.msu.edu/) against the Silva (SSU132) 16S rRNA database using a confidence threshold of 70% [50]. Random subsampling (rarefying) was applied to check for normality of raw data [51,52].

QIIME was used to calculate the alpha diversity (Ace, Chao, Shannon, Simpson and Sobs) for assessing the richness and diversity of the microbial community [53]. Paired t-test was applied to determine if differences in alpha diversity were significant. The Wilcoxon rank-sum test in STAMP was used to seek for significant differences between two samples, and the *p*-value was tested by Bonferroni correction with the threshold set at 0.05. Linear discriminant analysis (LDA) effect size (LEfSe) method was used to identify bacterial taxa with significant difference among groups (http://huttenhower.sph.harvard.edu/galaxy/root?tool_id=lefse_upload).

## 3. Results and discussions

Since the rewilding of the Przewalski’s horses in 2001, they have been suffering from severe natural horse botfly infestation. Yet the impact of this infection on their intestinal microbiota has never been addressed. Peachey et al. (2018) [42] indicated that changes of intestinal microbiota of infected horses after anthelmintic treatment were associated with the removal of parasites. Walshe et al. (2019) [54] investigated the effects of helminth infection on intestinal microbiota of horses by administration of anthelmintic drugs. The present study investigated the characteristics of the intestinal microbial community of the released Przewalski’s horses following and prior to their anthelmintic treatment to identify differences associated with horse botfly infestation. In the following results and discussions, abbreviations FATPH and PATPH are used for Przewalski’s horse following and prior to anthelmintic treatment, respectively.

### 3.1 Numeration of horse botfly larvae

Larvae of parasites in the feces of each FATPH collected in the three days following its ivermectin administration were counted with naked-eye inspection after morphological classification [16]. Second instar and third instar larvae were counted but only the third instars could be classified with their species. Horse botflies (*Gasterophilus* spp.) were the major parasites found in the FATPHs. The anthelmintic treatment flushed out a total of 12,154 larvae from the 7 FATPHs with an average of 1,736±755 per FATPH. This mean is lower than the 1904±536 observed from the digestive tracts of 6 dead Przewalski’s horses also at KNR by Huang et al. (2016) [16]. This is reasonable as the larvae collected in the present study were from feces rather than directly inside the horses. Hence horse-botfly infestation in the Przewalski’s horses at KNR is still severe. Among the total numbers of larvae collected in this study, 75.6% had already reached to their third instar stage. They were classified into 4 *Gasterophilus* spp., namely *G*. *pecorum*, *G*. *nigricornis*, *G*. *haemorrhoidalis*, and *G*. *intestinalis*. Their distributions are shown in Table 1. *G*. *pecorum* was the dominant species accounting for 91.8% of the total counts. The rest were shared by *G*. *nigricornis* (5.6%), *G*. *haemorrhoidalis* (2.4%), and *G*. *intestinalis* (0.2%). Although the second instar larvae could not be classified, it is believed that they should follow similar species distribution as the third instars. Similar *G*. *pecorum* species domination in the third instar larvae was also observed by Huang et al. (2016) [16].

**Table 1:** Cumulative totals of the third instar and second instar larvae of horse botfly in feces of FATPHs collected in the 3 days following ivermectin administration.

Sample	Third-instar species larva counts				Third-instar larva counts	Second-instar larva counts	Total larva counts
	*G*. *pecorum*	*G*. *nigricornis*	*G*. *haemorrhoidalis*	*G*. *intestinalis*			
FATPH1	642	12	52	1	707	17	724
FATPH2	1191	25	53	15	1284	135	1419
FATPH3	1494	355	19	0	1868	1098	2966
FATPH4	1607	43	39	1	1690	526	2216
FATPH5	908	75	18	1	1002	1126	2128
FATPH6	1905	4	0	0	1909	19	1928
FATPH7	686	1	35	2	724	49	773
Total	8433	515	216	20	9184	2970	12154

### 3.2 The intestinal microbial communities of FATPHs and PATPHs

With the development of molecular sequencing technology, DNA detection has become increasingly popular for studies in microbiology [55]. This technology was used to investigate the changes in the intestinal microbial community of the Przewalski’s horses under the main influence of *G*. *pecorum* infestation. The Illumina sequencing platform was used to process the 16S rRNA sequencing of the collected samples from both the FATPHs and PATPHs. A total of 33,8072,713 pairs of raw reads (FATPHs: 21,962,29±2,260,520; PATPHs: 26,333,802±1,002,001) and 814,650 valid sequences (FATPHs: 52,871±5,305; PATPHs: 63,508±2,618) were obtained from the 14 fecal samples. Results showed that the sequence length was mainly distributed between 400 bp and 440 bp with an average length of 415 bp. After checking for normality, the raw data were normalized for further analysis. Comparison of these two groups on their total numbers of valid bacterial sequences, pairs of raw reads, and the numbers of normalized OTUs that could be clustered into with a threshold of 97% homology similarity, together with the numbers of phyla, classes, orders, families, genera and species that the identified normalized OTUs could be assigned to is summarized in Table 2.

**Table 2:** Comparison of the group sequencing results between the FATPHs and PATPHs.

	Normalized OTU	Species	Genus	Family	Order	Class	Phylum
FATPHs	2148	449	250	109	62	32	22
Common	2047	426	238	106	62	32	22
PATPHs	2253	467	263	119	72	33	23

All numbers of the FATPHs are lower than those of the PATPHs in Table 2 with both sharing many. These indicate that the *G*. *pecorum* infestation in general increased slightly the complexity of the intestinal microbial community of the horses. As for the cumulative number without repetition of multiple samples, the FATPHs generated 2,148 OTUs from 7 samples, annotated to 22 phyla, 32 classes, 62 orders, 109 families, 250 genera, and 449 species, while the PATPHs generated 2,253 OTUs from 7 samples, annotated to 23 phyla, 33 classes, 72 orders, 119 families, 263 genera, and 467 species.

It is noted that both groups shared 22 bacterial phyla (Table 2) with the PATPHs having one additional phylum, *Gemmatimonadetes*. However, the relative abundance of *Gemmatimonadetes* in the PATPHs was extremely small (0.0006%). Reviewing results of individual samples found that it was only detected in one sample with the corresponding *Gemmatimonadetes*, *Gemmatimonadales*, *Gemmatimonadaceae* and *Gemmatimonas* found also in the same sample at the levels of class, order, family and genus, respectively. It is known that members of the *Gemmatimonadetes* phylum could be found in soil microbial communities but usually with low relative abundances [56], and its class *Gemmatimonadetes* is the dominant lineage mainly associated with soils [57]. Traces of this bacterial taxon found is suspected to be originated from the soil of the sampling site but not from the equine. Hence, results in Table 2 indicate that parasitic infestation affected richness of the horse intestinal microbial community at the level of order and lower.

**Fig 1:**
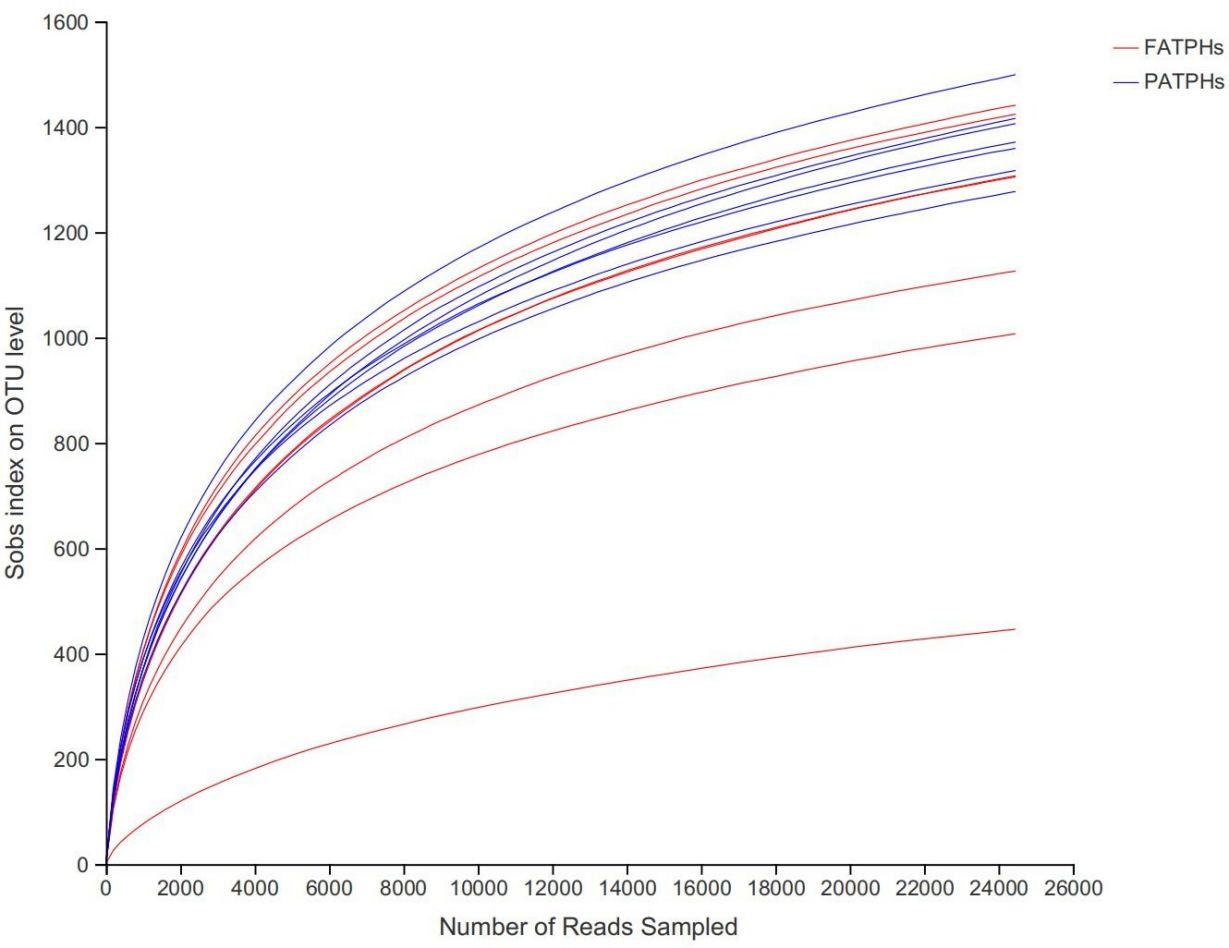
Rarefaction curves of normalized OTUs of 14 fecal samples obtained from the FATPHs and PATPHs.

The changes in alpha diversity metrics were then used to assess quantitatively the influence of *G*. *pecorum* infestation on diversity and richness of the microorganisms in the fecal samples of the FATPHs and PATPHs. This was done by comparing their Shannon and Simpson indexes that measure the diversity of their communities, and Ace, Chao and Sobs indexes that gauge their richness. Results are shown in Table 3. As for richness, the values of Sobs (FATPHs: 1151±348; PATPHs: 1378±72; *p* = 0.049), Ace (FATPHs: 1399.03±361.67; PATPHs: 1653.39±94.11; *p* = 0.047) and Chao (FATPHs: 1400.26±371.81; PATPHs: 1666.87±86.69; *p* = 0.038) indexes of the PATPHs were significantly higher than those of the FATPHs. The differences in Shannon (FATPHs: 5.00±1.35; PATPHs: 5.73±0.26) and Simpson indexes (FATPHs: 0.056±0.100; PATPHs: 0.016±0.013) between the two groups were found statistically insignificant (*p*>0.05), although values of the PATPHs in Table 3 showed a relatively slightly higher diversity than those of the FATPHs. The rarefaction curves of the two groups based on alpha diversity of Sobs [44] in Fig 1 show similar trend.

**Table 3:** Comparison of mean value of alpha diversity in indexes of Shannon, Simpson, Sobs, Ace and Chao between FATPHs and PATPHs.

	Community diversity		Community richness		
	Shannon	Simpson	Sobs	Ace	Chao
FATPHs	5.00±1.35	0.056±0.100	1151±348	1399.03±361.67	1400.26±371.81
PATPHs	5.73±0.26	0.016±0.013	1378±72	1653.39±94.11	1666.87±86.69
*p*-value	0.217	0.351	0.049	0.047	0.038

Previous studies indicated that variation in alpha diversity of the intestinal microbial community due to parasitic infestation could depend on the parasite type, the location of the intestines infected, and the host. For example, reduction of alpha diversity was found associated with *Schistosoma mansoni* infection in the lumina of mice [58], whilst no significant change of alpha diversity was seen in mice infected with *Metagonimus yokogawai* in cecum [59]. For pigs, Williams et al. (2017) [60] observed that an increase of alpha diversity was related with acute infection of *Ascaris suum* in their proximal colons. A trend towards an increased alpha diversity was observed in equines with high dose of cyathostomin infection in their intestines [42]. In addition, discrepancy of diversity could also be related with the phase of parasitic infection as reviewed by Peachey et al. (2017) [61] who suggested a decrease in the diversity of intestinal microbial community was possible during the initial parasite invasion of the gastrointestinal tract, and followed by a restoration (or increase) in the diversity upon the establishment of a chronic infection. Indexes of the FATPHs and PATPHs in the present study (Table 3 and Fig 1) indicate that the *G*. *pecorum* infestation increased significantly the richness but only suggested a possible diversity increasing trend of the intestinal microbial community of the Przewalski’s horses.

**Fig 2:**
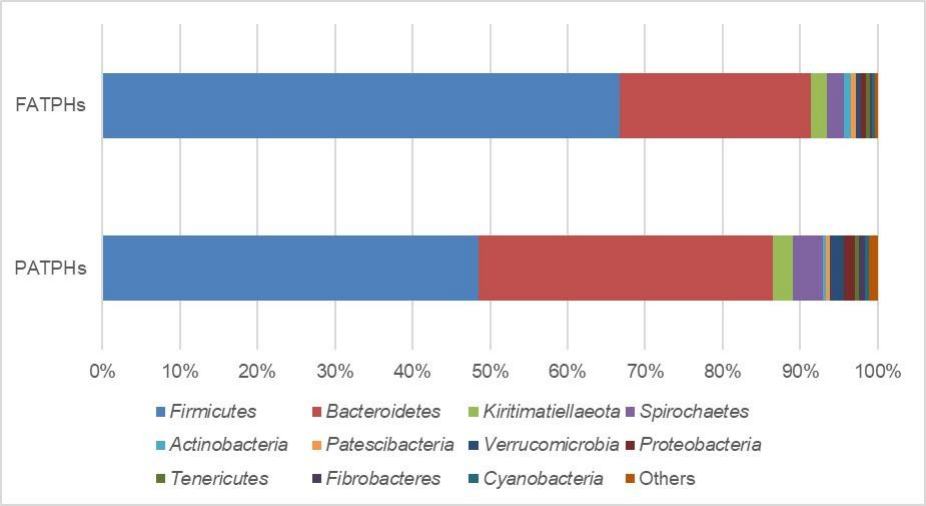
Top relative abundances of phyla ranked by -1 order of magnitude and higher based on the FATPHs with relative abundances of the corresponding phyla in the PATPHs shown. The relative abundances of the rest phyla were grouped as “Others”.

Differences in the microbial community structure and composition caused by *G*. *pecorum* infestation were further reviewed by the changes of relative abundance of various phyla in the fecal samples of the FATPHs and PATPHs as shown in Fig 2. Before addressing the changes, relative abundances of the prevalent phyla in PATPHs were compared with those at KNR reported in Li et al. (2019) [43] and in Tang et al. (2020) [44]. The two most abundant phyla in the PATPHs and in the other two studies were *Firmicutes* (PATPHs: 48.45%; Li et al. (2019) [43]: 51.81%, Tang et al. (2020) [44]: 35.00%) and *Bacteroidetes* (PATPHs: 38.04%; Li et al. (2019) [43]: 36.09%, Tang et al. (2020) [44]: 35.00%) with percentages at order of magnitude 1. The dominancy of these two phyla is consistent with studies of intestinal bacteria of horses [62] and other animals such as chickens [63], dholes [64], goats [65], pigs [66] and yaks [67].

Differences of the relative abundance of various phyla in the FATPHs and PATPHs shown in Fig 2 indicate that there were quite some changes for the two dominant phyla *Firmicutes* and *Bacteroidetes* following and prior to anthelmintic treatment. The 2 phyla took 91.36% (*Firmicutes*: 66.72%, *Bacteroidetes*: 24.64%) in the FATPHs and 86.49% (*Firmicutes*: 48.45%, *Bacteroidetes*: 38.04%) in the PATPHs, respectively. The presence of *G*. *pecorum* infestation decreased the relative abundance of *Firmicutes* largely by 18.27% (*p* = 0.041, S1 Table) and increased that of *Bacteroidetes* by 13.40% (*p* = 0.125, S1 Table). These results are consistent with study on helminth infected equines under anthelmintic treatment [54]. In terms of the *Firmicutes* to *Bacteroidetes* (F/B) ratio that could be positively correlated to body weight of horse [62], the PATPHs had an F/B ratio of 1.27 which is 0.47 times of the F/B (= 2.71) ratio of the FATPHs. It is suspected that the reduction of *Firmicutes* representation in the intestinal microbiota of the PATPHs due to *G*. *pecorum* infestation could decrease capacity of the microbiota to harvest energy leading to potential health or weight loss problems of the equines.

Meanwhile, the numbers of phyla with percentage of relative abundance at 0 order of magnitude were 2 in the FATPHs (*Kiritimatiellaeota*: 2.13%, *Spirochaetes*: 2.09%) and 4 in the PATPHs (*Spirochaetes*: 3.84%, *Kiritimatiellaeota*: 2.61%, *Verrucomicrobia*: 1.86%, *Proteobacteria*: 1.41%), respectively. At orders of magnitude of -1, -2, -3 and -4, the FATPHs had 7, 7, 3 and 1 phyla, respectively while the PATPHs had 9, 3, 3, and 2, respectively. Comparison of the relative abundances of the shared 22 phyla in the FATPHs and PATPHs shows that *G*. *pecorum* infestation increased the relative abundances of 15 phyla (*Bacteroidetes*, *Kiritimatiellaeota*, *Spirochaetes*, *Verrucomicrobia*, *Proteobacteria*, *Fibrobacteres*, *Cyanobacteria*, *Synergistetes*, *Epsilonbacteraeota*, *Elusimicrobia*, WPS-2, *unclassified_k__norank_d__Bacteria*, *Lentisphaerae*, *Planctomycetes*, *Fusobacteria*) and decreased those of the other 7 (*Firmicutes*, *Actinobacteria*, *Patescibacteria*, *Tenericutes*, *Armatimonadetes*, *Deferribacteres*, *Chloroflexi*).

At the genus level, both of the FATPHs and PATPHs had over 250 genera with slightly more in the PATPHs, and they shared 238 common genera. Reviewing the percentages of relative abundance at orders of magnitude showed that the FATPHs had 22, 59, 81, 78 and 10 genera present at orders of magnitude 0, -1, -2, -3, and -4, respectively while the PATPHs had 23, 56, 85, 83 and 16, respectively. There were 12 genera only found in the FATPHs and most were at -2 order of magnitude and lower except the unclassified_o_*Lactobacillales* which was at 0.29%. On the other hand, there were 25 genera existed only in the PATPHs and all were at -3 order of magnitude and lower. The similar uneven distribution of relative abundance across the genus spectrum in both of the FATPHs and PATPHs indicated that the *G*. *pecorum* infestation did not affect the diversity of the bacterial community much as shown by the alpha diversity indexes in Table 3.

**Fig 3:**
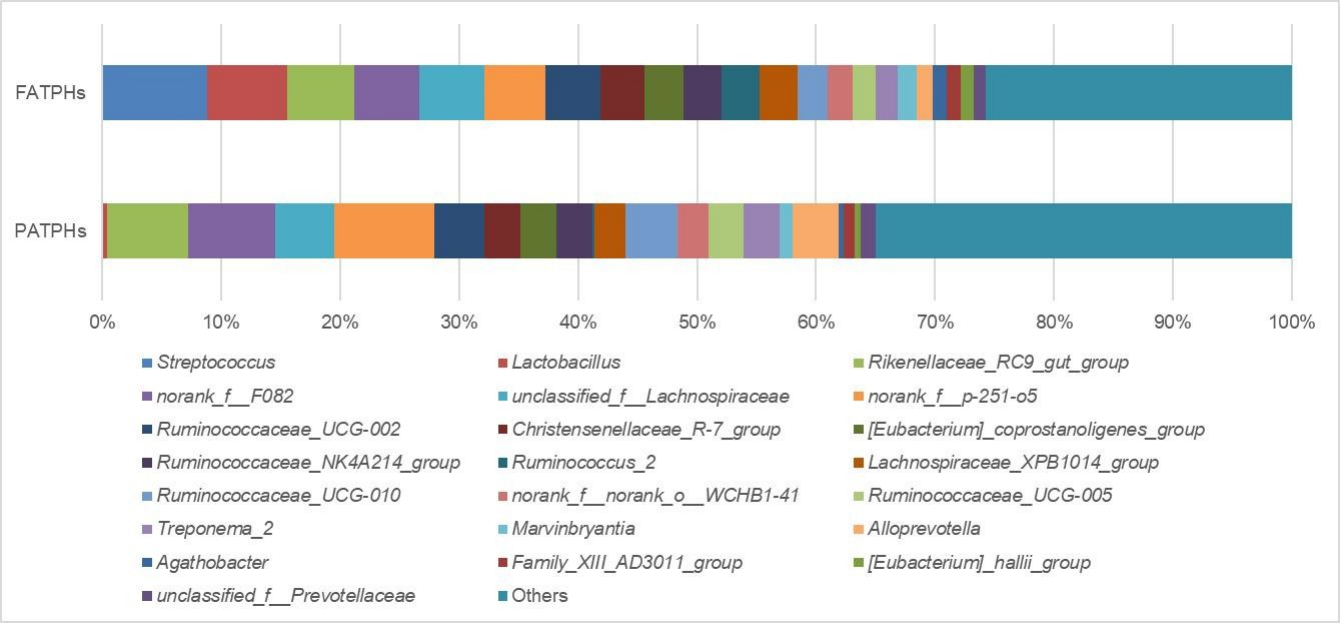
Top relative abundances of genera ranked by 0 order of magnitude and higher based on the FATPHs with relative abundances of the corresponding genera in the PATPHs shown. The relative abundances of the rest genera were grouped as “Others”.

Bar charts depicting the top relative abundance percentages ranked by 0 order of magnitude and higher based on the FATPHs are shown in Fig 3 together with relative abundances of the corresponding genera in the PATPHs. There were 22 genera in the FATPHs fell under this criteria, making up 74.23% of the total. The top 6 genera in the FATPHs with abundance higher than 5% were *Streptococcus* (8.79%), *Lactobacillus* (6.75%), *Rikenellaceae*_RC9_gut_group (5.68%), norank_f__F082 (5.47%), unclassified_f_*Lachnospiraceae* (5.43%), and *norank*_f_p-251-o5 (5.16%). Comparison of these 22 genera in the FATPHs with the corresponding ones in the PATPHs found that 13 of them had their abundances reduced totally by 22.81% and the others had theirs increased totally by 13.57% in the PATPHs. Hence there was a deficit of 9.24% relative abundance of the 22 genera in the PATPHs due to *G*. *pecorum* infestation. The major abundance reduction was with the top 2 (*Streptococcus* and *Lactobacillus*) and 11^th^ (*Ruminococcus*_2) genera in the FATPHs, having their abundances reduced by 8.73% (*Streptococcus*: FATPHs 8.79%, PATPHs 0.061%, *p* = 0.015, S2 Table), 6.41% (*Lactobacillus*: FATPHs 6.75%, PATPHs 0.34%, *p* = 0.041, S2 Table), and 3.08% (*Ruminococcus*_2: FATPHs 3.19%, PATPHs 0.11%, *p* = 0.073, S2 Table), respectively in the PATPHs. The other 9 genera in the FATPHs that had their relative abundances reduced in the PATPHs totally by 4.60%. On the other hand, 6 of the 9 genera in the FATPHs that showed growth of abundance in the PATPHs had over 1% increase. They, in the order of large to small increase, were *norank*_f__p-251-o5 (↑3.27%: FATPHs 5.16%, PATPHs 8.44%, *p* = 0.097, S2 Table), *Alloprevotella* (↑2.55%: FATPHs 1.33%, PATPHs 3.88%, *p* = 0.609, S2 Table), *Ruminococcaceae*_UCG-010 (↑1.93%: FATPHs 2.52%, PATPHs 4.45%, *p* = 0.041, S2 Table), *norank*_f__F082 (↑1.83%: FATPHs 5.47%, PATPHs 7.30%, *p* = 0.609, S2 Table), *Treponema*_2 (↑1.16%: FATPHs 1.80%, PATPHs 2.96%, *p* = 0.201, S2 Table), and *Rikenellaceae*_RC9_gut_group (↑1.15%: FATPHs 5.68%, PATPHs 6.84%, *p* = 0.609, S2 Table). The other 4 genera in the FATPHs only had their relative abundances increased by 1.69% totally in the PATPHs. Meanwhile, there were 3 other genera in the FATPHs with percentages of relative abundance at -1 order of magnitude had their abundances increased by more than 1% in the PATPHs. They were unclassified_o__*Bacteroidales* (↑1.32%: FATPHs 0.80%, PATPHs 2.12%, *p* = 0.125, S2 Table), *Phascolarctobacterium* (↑1.21%: FATPHs 0.31%, PATPHs 1.52%, *p* = 0.011, S2 Table), and *Akkermansia* (↑1.14%: FATPHs 0.66%, PATPHs 1.80%, *p* = 1.000, S2 Table). It appears that diversity at the genus level showed a partial increasing trend due to horse botfly infestation in the Przewalski’s horses. It was mainly through spreading the larger relative-abundance reduction (3.08% to 8.73%) of 3 prevailing genera to the smaller relative-abundance increase (1.15% to 3.27%) of 9 genera. However, these changes were not enough to affect significantly diversity of the overall bacterial community.

The major abundance reduction of the 2 most prevailing genera (*Streptococcus* and *Lactobacillus*) observed in the PATPHs, indicated that there might be changes of colic and digestion related conditions with the horses caused by their parasitic infestation. It is known that the relative abundance of *Streptococcus* in horses with a colic condition would increase [68]. The decrease of *Streptococcus* in the PATPHs due to *G*. *pecorum* infestation may reduce the chance of the horses experiencing colic. However, there are no literatures addressing the reduction of *Streptococcus* in parasite-infected equines until now. Further study is needed. Meanwhile, *Lactobacillus* are normally considered as lactic-producing bacteria [69]. Horses are hindgut fermenters that require large amounts of intestinal bacteria for converting food to short chain fatty acid, in which starch is fermented and converted to lactic acid by *Lactobacillus* [69]. In addition, *Lactobacillus* can also enhance the immune response of a host. Li et al. (2019) [43] suggested that Przewalski’s horses survived in the harsh natural environment with low quality diet had high abundance of *Lactobacillus* in their intestinal microbiota. Thus, the reduction of *Lactobacillus* to such low level in the PATPHs indicated that *G*. *pecorum* infestation could seriously affect the digestion ability and environmental adaptability of the Przewalski’s horses in the wild.

**Fig 4:**
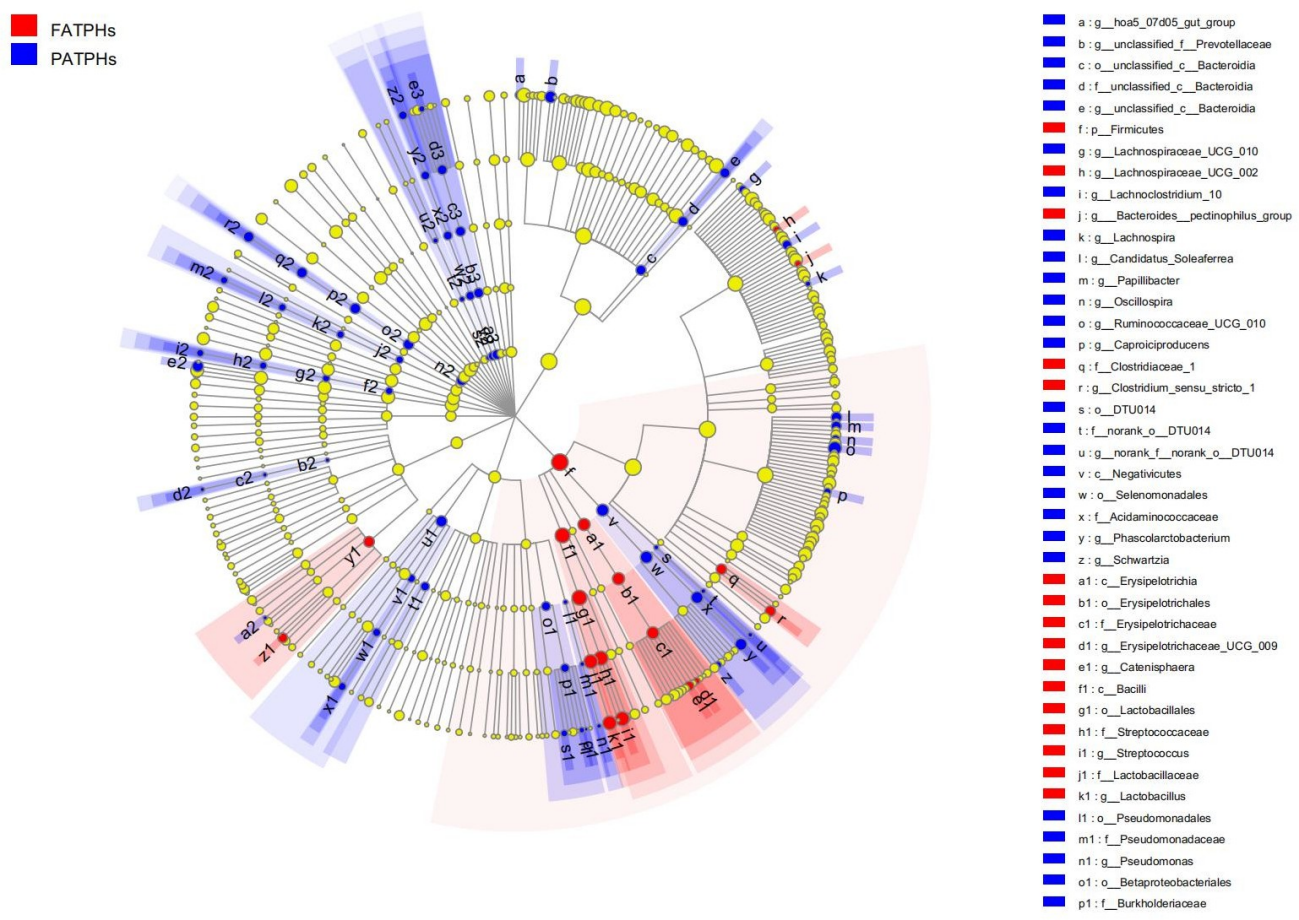
LEfSe cladogram showing a range of bacterial taxa at level of phylum to genus associated with the FATPHs (red) and PATPHs (blue) (α = 0.05, LDA > 2.0); the circle size represents the relative abundance of each taxon; the yellow circle indicates that the taxon has no significant difference between the FATPHs and PATPHs.

To identify the phylotypes with important functions that were responsible for the differences between the FATPHs and PATPHs, data of theirs were analyzed again with LEfSe. A total of 89 bacterial taxa were distinguished for the two horse groups, 18 for the FATPHs and 65 for the PATPHs (S1 Fig). The bacterial taxon with high LDA value (≥4.00) were genus *Ruminococcaceae*_UCG_010 (LDA = 4.00, *p* = 0.035) in the PATPHs, and order *Lactobacillales* (LDA = 4.91, *p* = 0.025), class *Bacilli* (LDA = 4.91, *p* = 0.035), phylum *Firmicutes* (LDA = 4.89, *p* = 0.035), family *Streptococcaceae* (LDA = 4.67, *p* = 0.013), genus *Streptococcus* (LDA = 4.66, *p* = 0.013), genus *Lactobacillus* (LDA = 4.53, *p* = 0.035), and family *Lactobacillaceae* (LDA = 4.53, *p* = 0.035) in the FATPHs (S3 Table). In addition, it was found that the functions of 1 class, 5 orders, 5 families, and 15 genera contributed greatly to the PATPHs, and those of 1 phylum, 2 classes, 2 orders, 4 families, and 7 genera to the FATPHs (Fig 4). For these bacterial taxon with high contribution, Wilcoxon rank-sum test with Bonferroni correction showed that the phylum *Firmicutes* (*p* = 0.041, S2A Fig), class *Bacilli* (*p* = 0.041, S2B Fig), order *Lactobacillales* (*p* = 0.030, S2C Fig), family *Streptococcaceae* (*p* = 0.015, S2D Fig), genus *Ruminococcaceae*_UCG_010 (*p* = 0.030, S2E Fig) and genus *Streptococcus* (*p* = 0.015, S2E Fig) had significant differences between the PATPHs and FATPHs. As the phylum *Firmicutes* and the genus *Streptococcus* showed significant differences between the PATPHs and FATPHs in both LEfSe and Wilcoxon rank-sum test, it confirms again that the effect of parasitic infestation on the digestive system of the Przewalski’s horses.

### 3.3 The distribution of pathobionts and symbionts

It was shown that there were major differences in the relative abundances of some health and digestion related genera in the intestinal microbiota of the Przewalski’s horses with and without *G*. *pecorum* infestation. It is believed that parasites are detrimental to the host [70] due to their impact on the groups of pathobionts and symbionts in the intestinal microbiota. Jenkins et al. (2018) [58] showed that the potentially pathogenic *Pseudomonas* and the lactic acid production related *Leuconostocaceae* in the human gut microbiome would decrease when infected by *Strongyloides stercoralis*. Gilchrist et al. (2016) [71] suggested that human infected with *Entamoeba histolytica* would lead to level expansion of the pathogenic *Prevotella*. Wu et al. (2012) [72] found that pigs infected by *Trichuris suis* had a reduction on their fibrolytic bacteria *Fibrobacter* and an augment of their pathogenic bacteria *Campylobacter*. Hence, a review on the abundance changes of genera known to be pathogenic or symbiotic between the FATPHs and PATPHs would provide another view on how *G*. *pecorum* infestation could affect the Przewalski’s horses.

**Fig 5:**
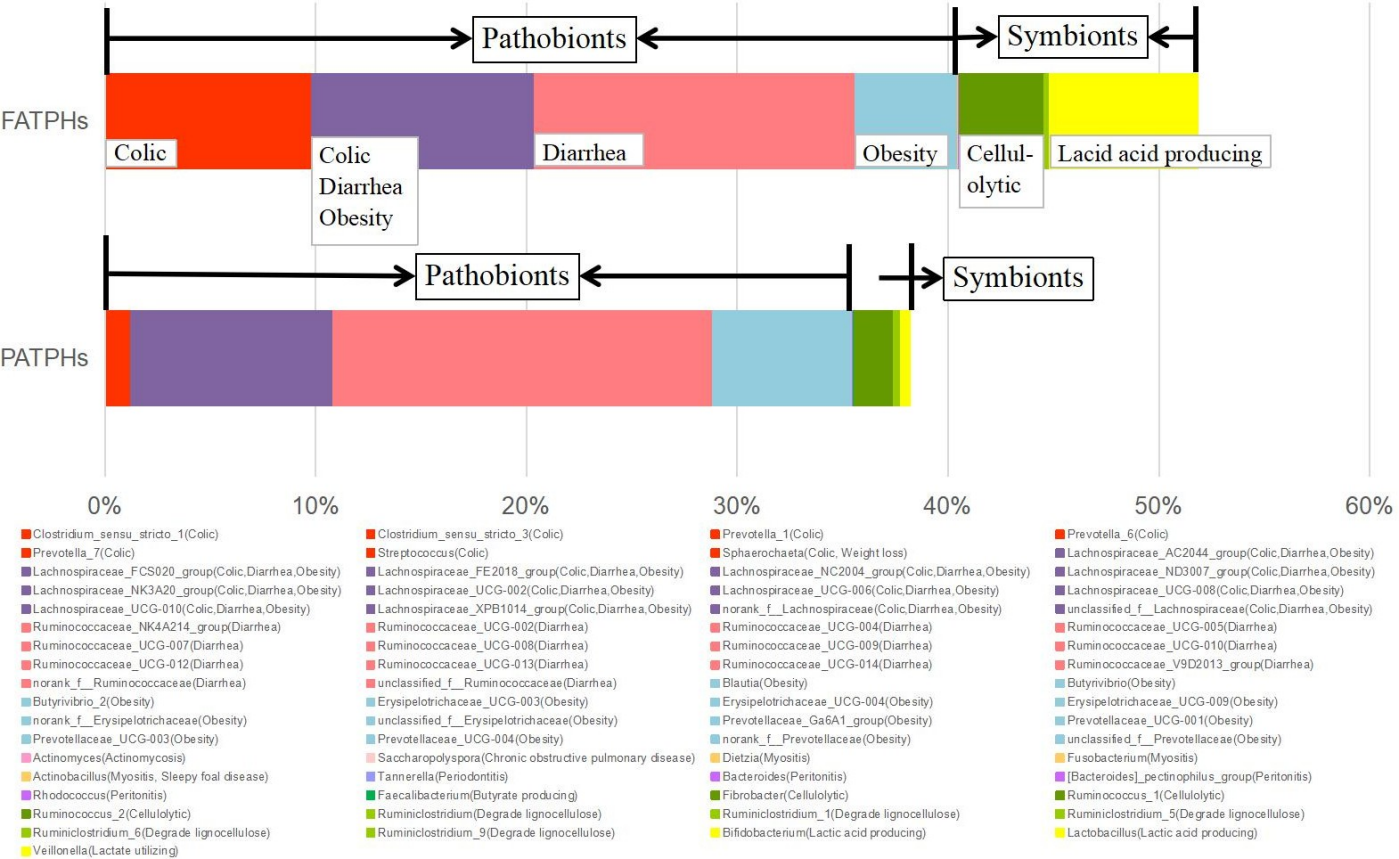
Relative abundances of pathobionts and symbionts at genus level in the FATPHs and PATPHs.

The pathogenic and symbiotic genera in the FATPHs and PATPHs identified through referencing published literatures are summarized in Table 4. There were 49 genera related to actinomycosis, chronic obstructive pulmonary disease, colic, diarrhea, myositis, obesity, periodontitis, peritonitis, sleepy foal disease, and weight loss in equines. Some genera were related to multiple illnesses, such as *Lachnospiraceae* was linked to colic, diarrhea and obesity. Meanwhile, there were 12 genera related to the digestive processes of equines that could be beneficial. They were associated with butyrate and lactic acid production, cellulolytic activity, lactate fermentation, and lignocellulose degradation. Bar charts showing the abundances of pathogenic and symbiotic genera in the FATPHs and PATPHs are presented in Fig 5. The total relative abundances of both genus groups in FATPHs were higher than those in the PATPHs. *G*. *pecorum* infestation decreased the abundances of both groups. Reduction of the pathogenic group was 4.92% (FATPHs 40.41%, PATPHs 35.49%, with PATPHs/FATPHs = 0.88) while that of the symbiotic group was larger at 8.63% (FATPHs 11.27%, PATPHs 2.64%, with PATPHs/FATPHs = 0.23). Ratios of the PATPHs to FATPHs abundance indicated the reduction of symbiotic group was relatively much more significant.

**Table 4:** Pathobionts and symbionts in FATPHs and PATPHs.

Type	Genus	Disease/function	References
Pathobionts	*Actinomyces*	Actinomycosis	[73]
	*Saccharopolyspora*	Chronic obstructive pulmonary disease	[74]
	*Clostridium_sensu_stricto*_1, *sensu_stricto*_3	Colic	[69]
	*Prevotella*_1, 6, 7	Colic	[68]
	*Streptococcus*	Colic	[68]
	*Sphaerochaeta*	Colic, Weight loss	[68,75]
	*Ruminococcaceae*_NK4A214_group, UCG-002, UCG-004, UCG-005, UCG-007, UCG-008, UCG-009, UCG-010, UCG-012, UCG-013, UCG-014, V9D2013_group	Diarrhea	[76]
	*Dietzia*	Myositis	[77]
	*Fusobacterium*	Myositis	[77]
	*Blautia*	Obesity	[62]
	*Butyrivibrio*, *Butyrivibrio*_2	Obesity	[62]
	*Erysipelotrichaceae*_UCG-003, UCG-004, UCG-009	Obesity	[62]
	*Prevotellaceae*_Ga6A1_group, UCG-001, UCG-003, UCG-004	Obesity	[62]
	*Lachnospiraceae_*AC2044_group, FCS020_group, FE2018_group, NC2004_group, ND3007_group, NK3A20_group, UCG-002, UCG-006, UCG-008, UCG-010, XPB1014_group	Obesity, Colic, Diarrhea	[62,68,76]
	*Bacteroides*	Peritonitis	[69]
	*Rhodococcus*	Peritonitis	[69]
	*Tannerella*	Periodontitis	[78]
	*Actinobacillus*	Sleepy foal disease, Myositis	[77,79]
Symbionts	*Faecalibacterium*	Butyrate production	[80]
	*Fibrobacter*	Cellulolytic activity	[80]
	*Ruminococcus*_1, _2	Cellulolytic activity	[69]
	*Veillonella*	Lactate fermentation	[69]
	*Bifidobacterium*	Lactic acid production	[69]
	*Lactobacillus*	Lactic acid production	[69]
	*Ruminiclostridium*, *Ruminiclostridium*_1, 5, 6, 9	Lignocellulose degradation	[81]

Reviewing the relative abundances of the pathogenic genera in the FATPHs by illness (including the genera associated with multiple illnesses) found that those related to diarrhea were the most abundant at 25.64%, followed by those linked to colic and obesity at 20.27% and 15.34%, respectively. It is noted that nearly half of the abundance (8.79%) of the colic related bacteria was from the *Streptococcus* genus. Meanwhile, genera associated with other illnesses were extremely scarce at a total of only 0.055%. Examining the relative abundances of the pathogenic genera in the PATPHs showed that those related to diarrhea continued to be the most abundant at 27.58% with an increase of 1.94% from that of the FATPHs. This increase was mainly by *Ruminococcaceae*_UCG-010 and *Ruminococcaceae*_UCG-005 which contributed 1.93% (*p* = 0.041, S2 Table) and 0.98% (*p* = 0.443, S2 Table), respectively. The obesity related genus group in the PATPHs became the second most abundant at 16.18% with a slight 0.84% increase from that of the FATPHs. Meanwhile, abundance of the colic related genus group dropped significantly (14.42%) from that of the FATPHs to 5.85% in the PATPHs. This large abundance reduction was mainly from *Streptococcus* which by itself reduced 8.73% (*p* = 0.015, S2 Table), almost all of its presence in the FATPHs. Meanwhile, other pathogenic genera in the PATPHs remained extremely scarce at a total abundance of 0.040%, a 0.015% reduction from that of the FATPHs.

Examining the 11.27% relative abundance of the symbiotic genera in the FATPHs found that those related to lactic acid production were the most abundant at 6.95%, followed by those linked to cellulolytic activity at 4.05%. It is noted that majority of the lactic acid production related bacteria was from *Lactobacillus* (6.75%), while the main contributor to the group associated with cellulolytic activity was *Ruminococcus*_2 (3.19%). The abundances of the other genus groups linked to lignocellulose degradation, lactate fermentation, and butyrate production were very low at 0.23%, 0.03% and 0.01% in the FATPHs, respectively. Cross-checking the relative abundances of these symbiotic genus groups with those in the PATPHs infested by *G*. *pecorum* showed that abundance of the lactic acid production genus group (*Lactobacillus* and *Bifidobacterium*) dropped significantly (6.61%, *Lactobacillus p* = 0.041, *Bifidobacterium p* = 1.000, S2 Table) from that of the FATPHs to 0.34%. Meanwhile, the abundance of the genus group, mainly from *Ruminococcus*_2, associated with cellulolytic activity also dropped relatively large by 3.08% (*p* = 0.073, S2 Table) from that (4.05%) of the FATPHs to 1.86%. As for the abundances of the other genus groups in the PATPHs linked to lignocellulose degradation, lactate fermentation, and butyrate production, their variations from those of the FATPHs were small with slight increases for those related to lignocellulose degradation (↑0.13%) and butyrate production (↑0.04%), and minimal decrease for that related to lactate fermentation (↓0.0023%).

It was found possible adverse impact of horse botfly infestation could be the significant reduction of *Lactobacillus* and *Bifidobacterium*, which are symbiotic genera related to lactic acid production. These two genera were previously found probiotics and were able to promote immunity against parasitic infection [82–84]. However, *Lactobacillus* has also been found positively correlated with the infection of *Trichuris muris* [85] and *Strongyloides venezuelensis* [86], opposite to the present observation with *G*. *pecorum*. Further studies are needed.

## 4. Conclusions

The problem of *G*. *pecorum* infestation in the rewilded Przewalski’s horses at KNR was found severe still. Its effects on their intestinal microbiota of the horses were estimated for the first time using high throughput sequencing on the fecal samples collected from 7 Przewalski’s horses following and prior to their anthelmintic treatment. The phylum *Firmicutes* and *Bacteroidetes* were found dominant in the Przewalski’s horses as in other animals. *G*. *pecorum* infestation decreased *Firmicutes* largely while it increased *Bacteroidetes*, causing the F/B ratio to reduce by over 50%. Indexes of alpha diversity indicated that *G*. *pecorum* infestation significantly increased the richness but not the diversity of the overall intestinal microbial community. Variations of genus relative abundance between the FATPHs and PATPHs showed a partial diversity increasing trend due to *G*. *pecorum* infestation. It was mainly through larger abundance reductions of the prevailing *Streptococcus*, *Lactobacillus* and *Ruminococcus*_2 together with smaller increases of other 9 genera. These were further confirmed by the significant differences observed in phylum *Firmicutes* and genus *Lactobacillus* and *Streptococcus* caused by *G*. *pecorum* infestation in the LEfSe and Wilcoxon rank-sum test. Meanwhile, reviewing the changes caused by *G*. *pecorum* infestation on relative abundance of the genera known pathogenic or symbiotic in published literatures suggested that potential negative impact on the wellbeing of the horses could be due to the significant reduction of the symbiotic *Lactobacillus* and *Bifidobacterium* that are related to lactic acid production. However, this was limited by the lack of knowledge on the actual functions of these symbiotic taxa. Metagenomics analysis is recommended to confirm this suggestion. It is noted that the present study was also statistically limited by the small size of samples due to the endangered status of the Przewalski’s horses, which could be improved over repeated studies in the future. Nonetheless, the present results still indicated that there were effects of the *G*. *pecorum* infestation on the intestinal microbiota of the Przewalski’s horses which could be harmful to them. Further studies are recommended.

## Supporting information

S1 Fig (TIF)

S2 Fig (TIF)

S1 Table (XLSX)

S2 Table (XLSX)

S3 Table (XLSX)

## References

[1] King, S.R.B., Boyd, L., Zimmermann, W., Kendall, B.E. *Equus ferus* (errata version published in 2016). The IUCN red list of threatened species 2015: e. T41763A97204950. 2015.

[2] Jiang Z., Zong H. 2019. Reintroduction of the Przewalski’s horse in China: status quo and outlook. Nature conservation research, 4:15–22.

[3] Gao X., Gu J., Zhou J. 1989. The change on the distribution area of the wild horse in the modern times. Arid zone research, 6:49–54.

[4] Xia C., Cao J., Zhang H., Gao X., Yang W., Blank D. 2014. *Reintroduction of Przewalski’s* horse (*Equus ferus przewalskii*) in Xinjiang, China: the status and experience. Biological conservation, 177, 142–147.

[5] Wakefield S., Knowles J., Zimmermann W., Van Dierendonck M.C., 2002. Status and action plan for the Przewalski’s horse (*Equus ferus przewalskii*). In: Moehlman P. (Ed.), Equids: Zebras, Asses and Horses. IUCN Publications Services Unit, Cambridge, UK, IUCN/SSC Equid Specialist Group, 82–92.

[6] Kaczensky P., Ganbaatar O., Wehrden H.V., Enksaikhan N., Lkhagvasuren D., Walzer C., 2007. Przewalski horse reintroduction in the great gobi B strictly protected area from species to ecosystem conservation. Mongolian journal of biological sciences. 5, 13–18. 10.22353/mjbs.2007.05.03 22064815 PMC3207201

[7] Duncan P. 1992. Zebras, asses, and horses: an action plan for the conservation of wild equids. IUCN Gland, Switzerland.

[8] Chen J., Weng Q., Chao J., Hu D., Taya K. 2008. Reproduction and development of the released Przewalski’s horses (*Equus przewalskii*) in Xinjiang, China. Journal of equine science, 19(1), 1–7. 10.1294/jes.19.1 24833949 PMC4019202

[9] Xu X., Huang Y., Hu J., Qi C. 1995. Parasites and their repelling of *Equus przewalskii* in Xinjiang. Chinese journal of veterinary medicine 7: 16. [In Chinese].

[10] Li K., Wu Z., Hu D., Cao J., Wang C. 2007. A report on new causative agent (*Gasterophilus* spp.) of the myiasis of Przewalski’s horse occurred in China. Chinese journal of animal and veterinary sciences: 38(8): 837–840. [In Chinese].

[11] Zhang H., Li K., Chen J., Hu D. 2007. Monitoring of *Equus przewalskii* parasites. Gansu animal and veterinary sciences 27: 94–96. [In Chinese].

[12] Liu S. 2012 Morphology and epidemiological investigation and the mitochondrial cytochrome oxidase I (COI) and 16S rRNA gene phylogenetic of *Gasterophilus*. Beijing Forestry University, Beijing.

[13] Wang W., Zhang D., Hu D., Chu H., Cao J., Ente M., et al. 2014. Population genetic structure of *Gasterophilus pecorum* in the Kalamaili Nature Reserve, Xinjiang, based on mitochondrial cytochrome oxidase (COI) gene sequence. Medical and veterinary entomology. 28 (S1): 75–82. 10.1111/mve.12073 25171609

[14] Liu S., Hu D., Li K. 2015. Oviposition site selection by Gasterophilus pecorum (Diptera: Gasterophilidae) in its habitat in Kalamaili Nature Reserve, Xinjiang, China. Parasite, 22: 34. 10.1051/parasite/2015034 26621549 PMC4664853

[15] Liu S., Li K., Hu D. 2016. The incidence and species composition of Gasterophilus (Diptera, *Gasterophilidae*) causing equine myiasis in northern Xinjiang, China. Veterinary parasitology, 217: 36–38. 10.1016/j.vetpar.2015.12.028 26827858

[16] Huang H., Zhang B., Chu H., Zhang D., Li K. 2016. *Gasterophilus* (*Diptera*, *gasterophilidae*) infestation of equids in the Kalamaili Nature Reserve, China. Parasite, 23,36. 10.1051/parasite/2016036 27593434 PMC5018932

[17] Zumpt F. 1965. Myasis in man and animals in the old world. Butterworts, London, UK. 10.1007/BF00329589

[18] Sequeira J., Tostes R., Oliveira‐Sequeira T. 2001. Prevalence and macro and microscopic lesions produced by Gasterophilus nasalis (Diptera: Oestridae) in the Botucatu Region, SP, Brazil. Veterinary parasitology, 102, 261–266. 10.1016/s0304-4017(01)00536-2 11777606

[19] Costa M. C., Weese J. S. 2018. Understanding the intestinal microbiome in health and disease. The veterinary clinics of North America. Equine practice, 34:1–12. 10.1016/j.cveq.2017.11.005 29402480

[20] Srivastava A., Lall R., Talukder J., DuBourdieu D., Gupta R.C. 2019. Iron transport tocopheryl polyethylene glycol succinate in animal health and diseases. Molecules, 24, 4289. 10.3390/molecules24234289 31775281 PMC6930530

[21] Julliand V., Grimm P. 2016. The microbiome of the horse hindgut: history and current knowledge. Journal of animal science, 94:2262–2274. 10.2527/jas.2015-0198 27285903

[22] Nicholson J. K., Holmes E., Kinross J., Burcelin R., Gibson G., Jia W., et al. 2012. Host-gut microbiota metabolic interactions. Science, 336:1262–1267. 10.1126/science.1223813 22674330

[23] Muegge B. D., Kuczynski J., Knights D., Clemente J. C., Gonzalez A., Fontana L., et al. 2011. Diet drives convergence in gut microbiome functions across mammalian phylogeny and within humans. Science, 332:970–974. 10.1126/science.1198719 21596990 PMC3303602

[24] Arumugam M., Raes J., Pelletier E., Paslier D.L., Yamada T., Mende D-R., et al. 2011. Enterotypes of the human gut microbiome. Nature, 473, 174–180. 10.1038/nature09944 21508958 PMC3728647

[25] Costea P. I., Hildebrand F., Manimozhiyan A., Bakhed F., Blaser M. J., Bushman F. D., et al. 2018. Enterotypes in the landscape of gut microbial community composition. Nature microbiology, 3(1): 8–16. 10.1038/s41564-017-0072-8 29255284 PMC5832044

[26] Sepulveda J., Moeller A.H. 2020. The effects of temperature on animal gut microbiomes. Frontiers in microbiology,11:384. 10.3389/fmicb.2020.00384 32210948 PMC7076155

[27] Nelson T. M., Rogers T. L., Brown M. V. 2013. The gut bacterial community of mammals from marine and terrestrial habitats. PLoS One, 8(12). 10.1371/journal.pone.0083655 24386245 PMC3875473

[28] Hong P. Y., Wheeler E., Cann I. K., Mackie R. I. 2011. Phylogenetic analysis of the fecal microbial community in herbivorous land and marine iguanas of the Galápagos Islands using 16S rRNA-based pyrosequencing. The ISME journal, 5(9), 1461–1470. 10.1038/ismej.2011.33 21451584 PMC3160690

[29] Waligora-Dupriet A. J., Lafleur S., Charrueau C. 2018. Head injury profoundly affects gut microbiota homeostasis: results of a pilot study. Nutrition, 45: 104–107. 10.1016/j.nut.2017.06.026 29129229

[30] Yoon M.Y., Yoon S.S. 2018. Disruption of the gut ecosystem by antibiotics. Yonsei medical journal, 59(1): 4–12. 10.3349/ymj.2018.59.1.4 29214770 PMC5725362

[31] Garrett L. A., Brown R., Poxton I. R. 2002. A comparative study of the intestinal microbiota of healthy horses and those suffering from equine grass sickness. Veterinary microbiology, 87:81–8. 10.1016/s0378-1135(02)00018-4 12079749

[32] Milinovich G.J., Burrell P.C., Pollitt C.C., Klieve A.V., Blackall L.L., Ouwerkerk D, et al. 2008. Microbial ecology of the equine hindgut during oligofructose-induced laminitis. The ISME journal, 2:1089. 10.1038/ismej.2008.67 18580970

[33] Costa M. C., Arroyo L. G., Allen-Vercoe E., Stämpfli H. R., Kim P. T., Sturgeon A., et al. 2012. Comparison of the fecal microbiota of healthy horses and horses with colitis by high throughput sequencing of the V3-V5 region of the 16S rRNA gene. PloS one, 7(7). 10.1371/journal.pone.0041484 22859989 PMC3409227

[34] Yoshida N., Yamashita T., Hirata K.I. 2018. Gut microbiome and cardiovascular diseases. Diseases. 6(3):56. 10.3390/diseases6030056 29966270 PMC6164700

[35] Frank D.N., St Amand A.L., Feldman R.A., Boedeker E.C., Harpaz N., Pace N.R. 2007. Molecular-phylogenetic characterization of microbial community imbalances in human inflammatory bowel diseases. Proceedings of the national academy of sciences. 104:13780–5. 10.1073/pnas.0706625104 17699621 PMC1959459

[36] Aydin O., Nieuwdorp M., Gerdes V. 2018. The gut microbiome as a target for the treatment of type 2 diabetes. Current diabetes reports. 18:55. 10.1007/s11892-018-1020-6 29931613 PMC6013535

[37] Scher J.U., Abramson S.B. 2011. The microbiome and rheumatoid arthritis. Nature reviews rheumatology. 7:569–578. 10.1038/nrrheum.2011.121 21862983 PMC3275101

[38] Zalar B., Haslberger A., Peterlin B. 2018. The role of microbiota in depression–a brief review. Psychiatria danubina. 30:136–41. 10.24869/psyd.2018.136 29930222

[39] Dart A. 2018. Gut microbiota bile acid metabolism controls cancer immunosurveillance. Nature reviews microbiology. 16:453. 10.1038/s41579-018-0053-9 29946123

[40] Weese J. S., Holcombe S. J., Embertson R. M., Kurtz K.A., Roessner H. A., Jalali M., et al. 2015. Changes in the faecal microbiota of mares precede the development of post partum colic. Equine veterinary journal, 47:641–649. 10.1111/evj.12361 25257320

[41] Clark A., Sallé G., Ballan V., Reigner F., Meynadier A., Cortet J., et al. 2018. Strongyle infection and gut microbiota: profiling of resistant and susceptible horses over a grazing season. Frontiers in physiology, 9:272. 10.3389/fphys.2018.00272 29618989 PMC5871743

[42] Peachey L.E., Molena R.A., Jenkins T.P., Cesare A.D., Traversa D., Hodgkinson J.E., et al. 2018. The relationships between faecal egg counts and gut microbial composition in UK Thoroughbreds infected by cyathostomins. International journal for parasitology, 48(6). 10.1016/j.ijpara.2017.11.003 29432771 PMC5946844

[43] Li Y., Zhang K., Liu Y., Li K., Hu D., Wronski T. 2019. Community composition and diversity of intestinal microbiota in captive and reintroduced Przewalski’s horse (*Equus ferus przewalskii*). Frontiers in microbiology, 10:1821. 10.3389/fmicb.2019.01821 31440229 PMC6693443

[44] Tang L., Li Y., Srivathsan A., Guo Y., Li K., Hu D., et al. 2020. Gut microbiomes of endangered Przewalski’s horse populations in short-and long-term captivity: implication for species reintroduction based on the soft-release strategy. Frontiers in microbiology, 11:363. 10.3389/fmicb.2020.00363 32226419 PMC7081077

[45] Russell W. M. S., Burch R. 1959. The Principles of humane experimental technique. London, UK: Methuen.

[46] John K., Leon A. 2003. Mechanosensory inputs influence *Caenorhabditis elegans* pharyngeal activity via ivermectin sensitivity genes. Genetics,164(1):153–62. 12750328 10.1093/genetics/164.1.153 PMC1462566

[47] Martin R. J., Robertson A. P., Choudhary S. 2020. Ivermectin: an anthelmintic, an insecticide, and much more. Trends in parasitology, 37, 1. 10.1016/j.pt.2020.10.008 33189582 PMC7853155

[48] Kunz I. G., Reed K. J., Metcalf J. L., Hassel D. M., Coleman R. J., Hess T. M., et al. 2019. Equine fecal microbiota changes associated with anthelmintic administration. Journal of equine veterinary science, 77, 98–106. 10.1016/j.jevs.2019.01.018 31133326

[49] Riquelme J., Cazanga V., Jeldres J., Pérez R. 2018. Pharmacokinetics of ivermectin in sheep following pretreatment with *Escherichia coli* endotoxin. Journal of veterinary pharmacology and therapeutics. 41(2). 10.1111/jvp.12665 29889311

[50] Katherine R. A., Carl J. Y., Angela K. 2013. Habitat degradation impacts black howler monkey (*Alouatta pigra*) gastrointestinal microbiomes. The ISME Journal. 7,1344–1353. 10.1038/ismej.2013.16 23486247 PMC3695285

[51] Salem S.E., Maddox T.W., Berg A., Antczak P., Ketley J.M., Williams N.J., et al. 2018. Variation in faecal microbiota in a group of horses managed at pasture over a 12-month period. Scientific reports. 8, 8510. 10.1038/s41598-018-26930-3 29855517 PMC5981443

[52] Lin H., Peddada S.D. 2020. Analysis of microbial compositions: a review of normalization and differential abundance analysis. NPJ biofilms and microbiomes 6, 60. 10.1038/s41522-020-00160-w 33268781 PMC7710733

[53] Caporaso J. G., Kuczynski J., Stombaugh J., Bittinger K., Bushman F.D., Costello E.K., et al. 2010. QIIME allows analysis of high-throughput community sequencing data. Nature methods, 7:335. 10.1038/nmeth.f.303 20383131 PMC3156573

[54] Walshe N., Duggan V., Cabrera-Rubio R., Crispie F., Cotter P., Feehan O., et al. 2019. Removal of adult cyathostomins alters faecal microbiota and promotes an inflammatory phenotype in horses. International journal for parasitology. 49,6, 489–500. 10.1016/j.ijpara.2019.02.003 30986403

[55] Jovel J., Patterson J., Wang W., Hotte N., O’Keefe S., Mitchel T., Wong G. K. S. 2016. Characterization of the gut microbiome using 16S or shotgun metagenomics. Frontiers in microbiology, 7, 459. 10.3389/fmicb.2016.00459 27148170 PMC4837688

[56] DeBruyn J. M., Nixon L. T., Fawaz M. N., Johnson A. M., Radosevich M. 2011. Global biogeography and quantitative seasonal dynamics of *Gemmatimonadetes* in soil. Applied and environmental microbiology, 77:6295–6300. 10.1128/AEM.05005-11 21764958 PMC3165389

[57] Hanada S., Sekiguchi Y. 2014. The phylum *Gemmatimonadetes*. In the Prokaryotes. Springer, 4th edition, 11, 677–681.

[58] Jenkins T.P., Peachey L.E., Ajami N.J., MacDonald A.S., Hsieh M.H., Brindley P.J., et al. 2018. *Schistosoma mansoni* infection isassociated with quantitative and qualitative modifcations of the mammalian intestinal microbiota. Scientific reports, 8, 1:12072–018. 10.1038/s41598-018-30412-x 30104612 PMC6089957

[59] Kim J., Kim E-M., Yi M., Lee J., Lee S., Hwang Y., et al. 2018. Intestinal fluke *Metagonimus yokogawai* infection increases probiotic Lactobacillus in mouse cecum. Experimental parasitology, 193, 45–50. 10.1016/j.exppara.2018.08.002 30149004

[60] Williams A.R., Krych L., Fauzan Ahmad H., Nejsum P., Skovgaard K., Nielsen D.S., et al. 2017. A polyphenol-enriched diet and Ascaris suum infection modulate mucosal immune responses and gut microbiota composition in pigs. PLoS one, 12(10): e0186546. 10.1371/journal.pone.0186546 29028844 PMC5640243

[61] Peachey L.E., Jenkins T.P., Cantacessi C. 2017. This gut ain’t big enough for both of us. Or is it? Helminth-microbiota interactions in veterinary species. Trends in parasitology, 33, 619–632. 10.1016/j.pt.2017.04.004 28506779

[62] Biddle A., Tomb J-F., Fan Z. 2018. Microbiome and blood analyte differences point to community and metabolic signatures in lean and obese horses. Frontiers in veterinary science, 5:225. 10.3389/fvets.2018.00225 30294603 PMC6158370

[63] Xu Y., Yang H., Zhang L., Su Y., Shi D., Xiao H., et al. 2016. High-throughput sequencing technology to reveal the composition and function of cecal microbiota in Dagu chicken. BMC microbiology, 16(1), 259. 10.1186/s12866-016-0877-2 27814685 PMC5097418

[64] Wu X., Zhang H., Chen J., Shang S., Wei Q., Yan J., et al. 2016. Comparison of the fecal microbiota of dholes high-throughput Illumina sequencing of the V3–V4 region of the 16S rRNA gene. Applied microbiology and biotechnology, 100(8), 3577–3586. 10.1007/s00253-015-7257-y 26728019

[65] Han X., Yang Y., Yan H., Wang X., Qu L., Chen Y. 2015. Rumen bacterial diversity of 80 to 110-day-old goats using 16S rRNA sequencing. PloS one, 10(2). 10.1371/journal.pone.0117811 25700157 PMC4336330

[66] Kim H. B., Isaacson R. E. 2015. The pig gut microbial diversity: understanding the pig gut microbial ecology through the next generation high throughput sequencing. Veterinary microbiology, 177(3–4), 242–251. 10.1016/j.vetmic.2015.03.014 25843944

[67] Guo W., Li Y., Wang L., Wang J., Xu Q., Yan T., et al. 2015. Evaluation of composition and individual variability of rumen microbiota in yaks by 16S rRNA high-throughput sequencing technology. Anaerobe, 34, 74–79. 10.1016/j.anaerobe.2015.04.010 25911445

[68] Stewart H. L., Southwood L. L., Indugu N., Vecchiarelli B., Engiles J. B., Pitta D. 2018. Differences in the equine faecal microbiota between horses presenting to a tertiary referral hospital for colic compared to an elective surgical procedure. Equine veterinary journal, 51(3). 10.1111/evj.13010 30153353

[69] Dicks L. M. T., Botha M., Dicks E., Botes M. 2014. The equine gastro-intestinal tract: an overview of the microbiota, disease and treatment. Livestock science, 160, 69–81.

[70] Cortes A., Rooney J. J., Bartley D. J., Nisbet A., Cantacessi C. 2020. Helminths, host, and their microbiota: new avenues for managing gastrointestinal helminthiases in ruminants. Expert review of anti-infective therapy, 18:10, 977–985. 10.1080/14787210.2020.1782188 32530331

[71] Gilchrist C.A., Potri S.E., Schneider B.N., Reichman D.J., Jiang N., Begum S., et al. 2016. Role of the gut microbiota of children in diarrhea due to the protozoan parasite *Entamoeba histolytica*. The journal of infectious diseases, 213, 1579–1585. 10.1093/infdis/jiv772 26712950 PMC4837909

[72] Wu S., Li R.W., Li W., Beshah E., Dawson H.D., Urban J.F. 2012. Worm burden-dependent disruption of the porcine colon microbiota by *Trichuris suis* infection. PLoS One, 74:e35470. 10.1371/journal.pone.0035470 22532855 PMC3332011

[73] Murakami S., Kobayashi T., Sekigawa Y., Torii Y., Kanesaki Y., Ishige T., et al. 2018. *Actinomyces denticolens* as a causative agent of actinomycosis in animals. The journal of veterinary medical science, 80(11):1650–1656. 10.1292/jvms.18-0207 30224576 PMC6261818

[74] Marinkovic D. 2007. Cellular basis of chronic obstructive pulmonary disease in horses. International review of cytology, 257, 213–247. 10.1016/S0074-7696(07)57006-3 17280899

[75] Morrison P., Newbold C.J., Jones E., Worgan H.J., Grove-White D.H., Dugdale A.H., et al. 2020. The equine gastrointestinal microbiome: Impacts of weight-loss. BMC veterinary research, 2,16838. 10.1186/s12917-020-02295-6 32131835 PMC7057583

[76] Schoster A., Staempfli H.R., Guardabassi L.G., Jalali M., Weese J.S. 2017. Comparison of the fecal bacterial microbiota of healthy and diarrheic foals at two and four weeks of life, BMC veterinary research, 13, 1. 10.1186/s12917-016-0931-1 28558788 PMC5450145

[77] Rifici C., Attili A-R., De Biase D., Gonçalves dos Santos R., Seyffert N., De Paula Castro T. L., et al. 2020. Atypical multibacterial granulomatous myositis in a horse: first report in Italy. Veterinary sciences, 7(2), 47. 10.3390/vetsci7020047 PMC7355418 32326275

[78] Sykora S., Pieber K., Simhofer H., Hackl V., Brodesser D., Brandt S. 2013. Isolation of *Treponema* and *Tannerella* spp. from equine odontoclastic tooth resorption and hypercementosis related periodontal disease. Equine veterinary journal, 46(3), 358–363. 10.1111/evj.12115 23742079

[79] Christiane M., Marcel S., Kristin K. 2020. Detection of *Actinobacillus equuli* ssp. *equuli* in piglets with purulent polyarthritis and tendovaginitis. Tieraerztliche Praxis Ausgabe Grosstiere Nutztiere, 48,1,51–58. 10.1055/a-1067-3908 32059237

[80] Kobayashi R., Nagaoka K., Nishimura N., Koike S., Takahashi E., Niimi K., et al. 2020. Comparison of the fecal microbiota of two monogastric herbivorous and five omnivorous mammals. Animal science journal, 91(1). 10.1111/asj.13366 32285557 PMC7216987

[81] Sheng T., Zhao L., Gao L-F., Liu W-Z., Cui M-H., Guo Z-C., et al. 2016. Lignocellulosic saccharification by a newly isolated bacterium, *Ruminiclostridium thermocellum* M3 and cellular cellulase activities for high ratio of glucose to cellobiose. Biotechnology for biofuels, 9(1). 10.1186/s13068-016-0585-z 27525041 PMC4982309

[82] Mukhopadhyay B., Ganguly N.K. 2014. The unexplored role of probiotics on the parasitic pathogens. Food and nutrition sciences. 5, 2177–2184.

[83] Travers M., Florent I., Kohl L., Grellier P. 2011. Probiotics for the control of parasites: an overview. Journal of parasitology research. 11, 610769. 10.1155/2011/610769 21966589 PMC3182331

[84] Dalloul R.A., Lillehoj H.S., Shellem T.A., Doerr J. A. 2003. Enhanced mucosal immunity against *Eimeria acervulina* in broilers fed a *Lactobacillus*-based probiotic. Poultry science, 82, 62–66. 10.1093/ps/82.1.62 12580246

[85] Holm J. B., Sorobetea D., Kiilerich P., Ramayo-Caldas Y., Estelle J., Ma T., et al. 2015. Chronic Trichuris muris infection decreases diversity of the intestinal microbiota and concomitantly increases the abundance of Lactobacilli. PLoS one, 10, e0125495. 10.1371/journal.pone.0125495 25942314 PMC4420551

[86] Pace F., Carvalho B.M., Zanotto T.M., Santos A., Guadagnini D., Silva K.L.C, et al. 2018. Helminth infection in mice improves insulin sensitivity via modulation of gut microbiota and fatty acid metabolism. Pharmacological research, 132, 33–46. 10.1016/j.phrs.2018.04.008 29653264

