# Supplementary figures and images for: Effects of Gasterophilus pecorum infestation on the intestinal microbiota of the rewilded Przewalski’s horses in China
Source: PLoS One. 2021 May 11;16(5):e0251512. doi: 10.1371/journal.pone.0251512 (PMC8112688; doi:10.1371/journal.pone.0251512)

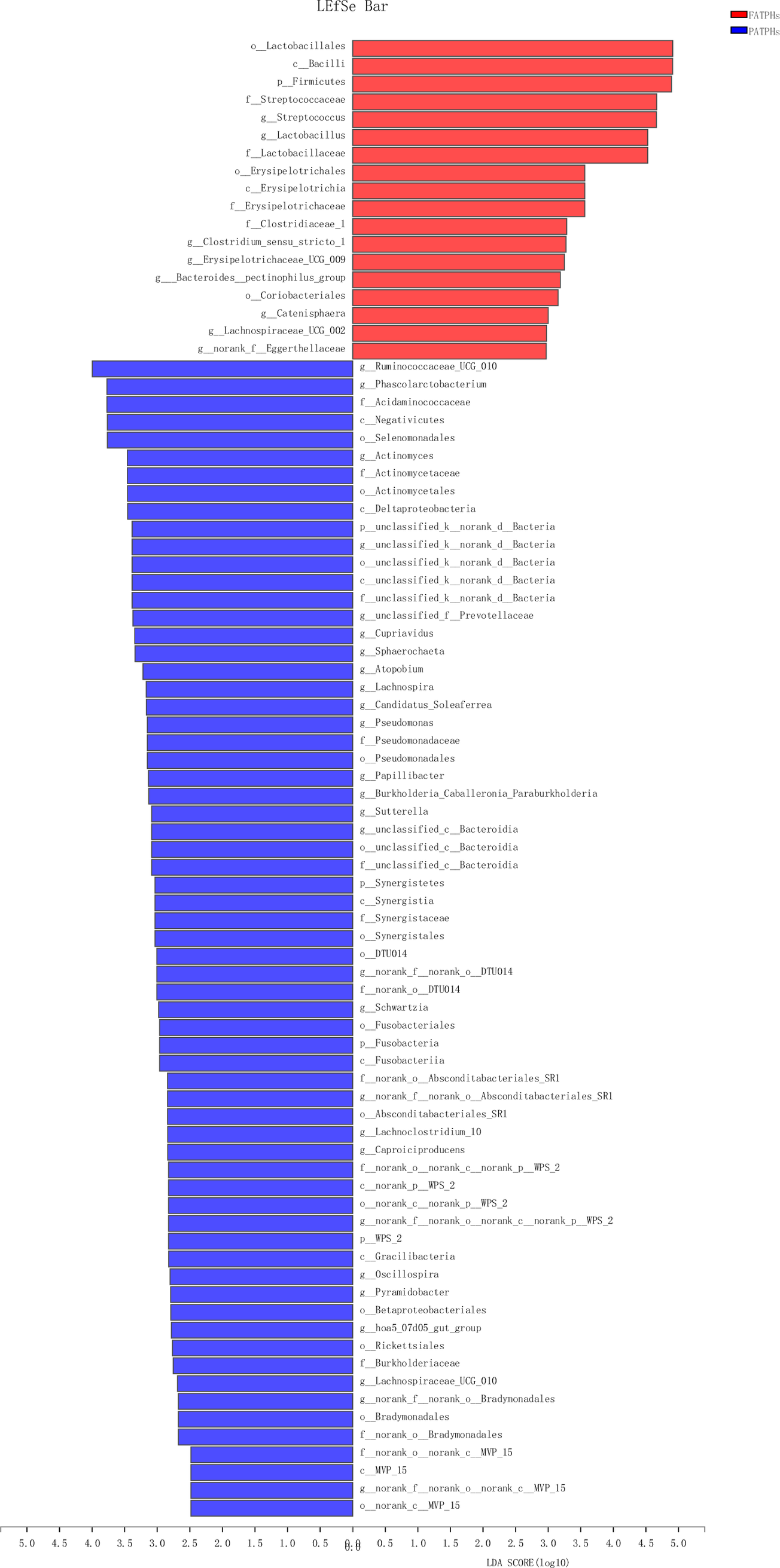

Supplement: S1 Fig — (TIF) [file pone.0251512.s001.tif]

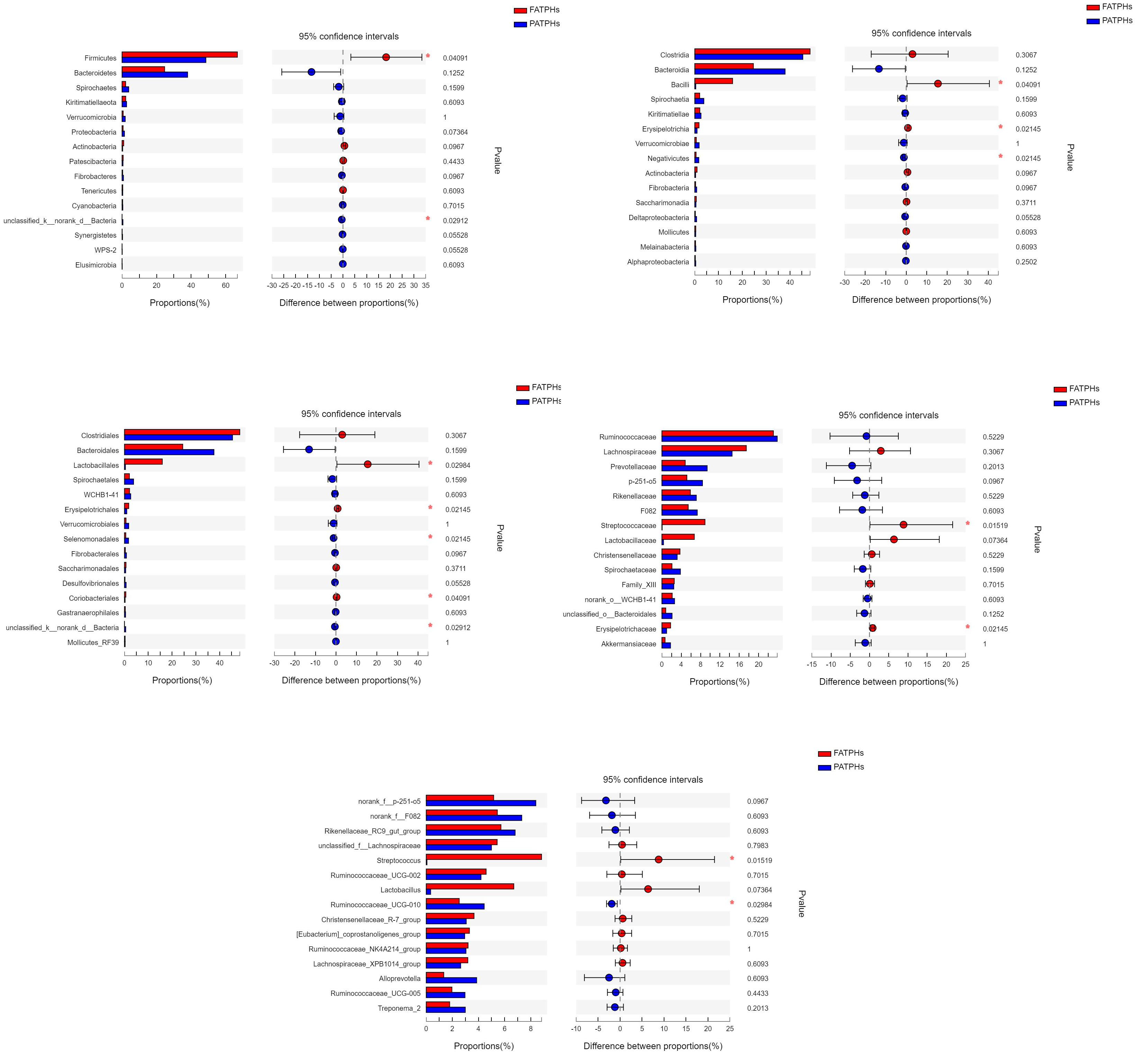

Supplement: S2 Fig — (TIF) [file pone.0251512.s002.tif]
